# Self-Aligned Bilayers for Flexible Free-Standing Organic Field-Effect Transistors

**DOI:** 10.1021/acsami.1c15208

**Published:** 2021-12-04

**Authors:** Hanna Zajaczkowska, Lothar Veith, Witold Waliszewski, Malgorzata A. Bartkiewicz, Michal Borkowski, Piotr Sleczkowski, Jacek Ulanski, Bartlomiej Graczykowski, Paul W. M. Blom, Wojciech Pisula, Tomasz Marszalek

**Affiliations:** † Department of Molecular Physics, Faculty of Chemistry, Lodz University of Technology, Zeromskiego 116, 90-924 Lodz, Poland; ‡ Max Planck Institute for Polymer Research, Ackermannweg 10, 55128 Mainz, Germany; § Faculty of Physics, Adam Mickiewicz University, Uniwersytetu Poznanskiego 2, 61-614 Poznan, Poland

**Keywords:** organic semiconductor, semiconductor/dielectric blend, self-aligned bilayer, field-effect transistors, trap-free interface, flexible free-standing transistor

## Abstract

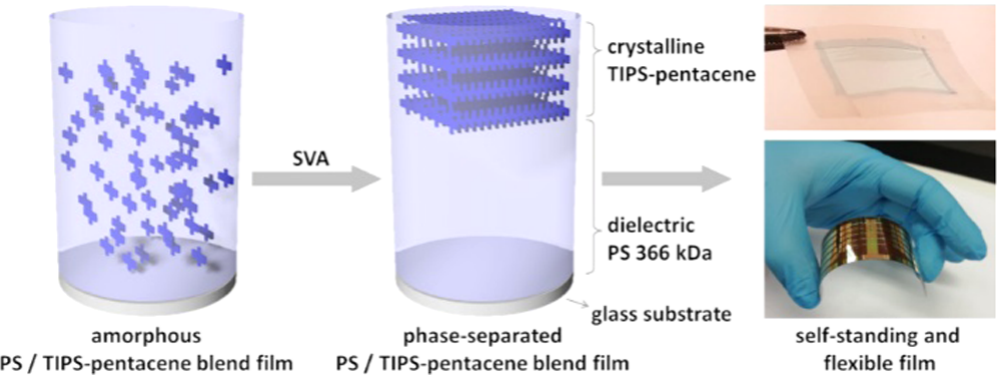

Free-standing and flexible field-effect transistors based on 6,13-bis(triisopropylsilylethynyl)-pentacene (TIPS-pentacene)/polystyrene bilayers are obtained by well-controlled phase separation of both components. The phase separation is induced by solvent vapor annealing of initially amorphous blend films, leading to crystallization of TIPS-pentacene as the top layer. The crystallinity and blend morphology strongly depend on the molecular weight of polystyrene, and under optimized conditions, distinct phase separation with a well-defined and trap-free interface between both fractions is achieved. Due to the distinct bilayer morphology, the resulting flexible field-effect transistors reveal similar charge carrier mobilities as rigid devices and additionally pronounced environmental and bias stress stabilities. The performance of the flexible transistors remains stable up to a strain of 1.8%, while above this deformation, a close relation between current and strain is observed that is required for applications in strain sensors.

## Introduction

Solution-processable organic semiconductors offer great advantages for thin-film processing of mechanically flexible electronic devices.^1−5^ The processing conditions determine to a great extent the molecular organization that is important for the charge carrier transport.^6−8^ A high molecular order in large domains ensures an unhindered transport of charge carriers, especially in organic field-effect transistors (OFETs).^8−12^ As a great advantage, the relatively low elastic modulus of organic semiconductors compared to their inorganic counterparts permits certain stretching and bending of the devices without serious degradation of the electrical performance within the mechanical properties of the active material.^13−16^ Flexible OFETs also require plastic substrates, which, however, show disadvantages. Their high surface roughness typically reduces the molecular order in the semiconducting film and therefore the device performance.^17,18^ In addition, a dielectric layer is needed to separate the gate electrode from the semiconductor. Finally, the choice of processing solvents for the semiconductor is limited to avoid damage of the thermoplastic substrate during the solution deposition of the active film.^19,20^

An alternative strategy to circumvent the solution deposition on plastic substrates is based on free-standing films obtained from organic semiconductor/insulating polymer blends. The idea of crystallization of small molecules in an insulating polymer matrix was introduced already in the 80s as the so-called “reticulate doping”^21,22^ that allowed creating conducting composites with percolation thresholds below 1 wt %. Another modification was the “two-step reticulate doping”, in which the as-cast blend film is exposed to solvent vapor using a good solvent for the polymer, but rather poor ones for the dispersed small molecules.^23^ Solvent vapor penetrates the polymer film and induces diffusion, nucleation, and subsequent crystallization of the small molecules in the blend film. This method is today commonly referred to as solvent vapor annealing (SVA). But so far, such binary blends have been mainly applied to improve the semiconductor morphology.^24−27^ The phase separation of the two components is determined by the change of Gibbs free energy of mixing (Δ*G*_m_) and the interaction parameter χ from the Flory–Huggins theory.^28^ Both factors are controlled by a proper choice of components, blending ratio, and processing conditions to adjust favorable phase separation with the desired blend morphology.^29^ One important factor among others is the molecular weight (*M*_w_) of the insulating polymer. The entropy of mixing is decreased and Δ*G*_m_ is increased for high-*M*_w_ insulators. For this reason, blends of high-*M*_w_ poly(α-methylstyrene) (PαMS) and 6,13-bis(triisopropylsilylethynyl)-pentacene (TIPS-pentacene) tend to phase-separate in thin films, while both components are homogeneously intermixed in the case of low-*M*_w_ PαMS.^30^ The calculation of Δ*G*_m_ is also valuable to estimate the optimal blend ratio for efficient phase separation. Calculations for blends of TIPS-pentacene and poly[bisphenol A carbonate-co-4,40-(3,3,5-trimethylcyclohexy-lidene)diphenol carbonate] indicated an optimum ratio of 1:4 that was experimentally confirmed for phase-separated blend films with high charge carrier mobilities.^31^ Besides the impact of Δ*G*_m_ on the phase separation, the resulting blend morphology in thin films is governed by solute–solvent, solute–substrate, and solute–solute interactions that are also determined by the blend composition, processing conditions, and surface energy of the substrate.^32^ Blending of crystalline small-molecule semiconductors with an insulating polymer improves their surface wettability during processing and crystallinity in the final film, typically combined with vertical phase separation as required for an unhindered charge carrier transport in the in-plane direction of OFETs.^33−35^ One reason for the higher crystallinity of the semiconducting phase is an extended drying time during solvent evaporation in the presence of the insulating polymer. In the vertically phase-separated bilayer morphology, the organic semiconductor can phase-separate as a top layer with the insulating layer serving as an underneath dielectric or as a bottom layer with protecting insulating encapsulation.^35,36^ The mechanism of the vertical/lateral phase separation and the crystallization process has been widely discussed in the literature.^37,38^ It has been concluded that the composite morphology is an outcome of various physicochemical factors that occur during film deposition and solidification. Depending on the blend components, deposition technique, and posttreatment conditions, lateral or vertical phase separation arises.^39−41^ In parallel to the phase separation, solidification occurs according to three possible scenarios: (i) crystallization (nucleation and growth) and (ii) binodal or (iii) spinodal decomposition.^42,43^ Both nucleation and crystallization mechanisms highly depend on the applied temperature.^44^ High temperature increases the evaporation rate and enhances the formation of nuclei and finally the phase separation. In addition, solute–solvent, solute–substrate, and solute–solute interactions may decide about the phase separation and blend morphology. From the application point of view, such as flexible electronic circuits, sensors, or memories, bilayer blends are worth further investigation.^45^

Due to the lower Young’s modulus of the thermoplastic insulating polymers, the organic semiconductor blend films are beneficial for flexible electronic applications.^46,47^ Especially, free-standing bilayer films are attractive due to their simplified fabrication process when the bottom insulating polymer layer is exploited as a dielectric and substrate at the same time. This setup additionally allows the reduction of the overall device thickness that is required for applications as electronic skin. So far, free-standing bilayer OFETs have been only reported for poly(3-hexylthiophene), reaching a charge carrier mobility of 0.02 cm^2^ V^–1^ s^–1^.^48^ Due to their high crystallinity, small-molecule semiconductors might be more attractive candidates to achieve higher device performance when their processing into homogeneous films and high rigidity and fragility are controlled.

In this work, TIPS-pentacene/polystyrene (PS) flexible and free-standing bilayer OFETs have been fabricated following systematic optimization of the *M*_w_ of polystyrene (PS) and the processing conditions. The resulting OFETs reveal a maximum charge carrier mobility of 0.4 cm^2^ V^–1^ s^–1^ and pronounced environmental and bias stress stability. The excellent device characteristics found for PS with a *M*_w_ of 366 kDa are attributed to well-defined phase separation with large and highly crystalline TIPS-pentacene domains in the top layer. The close relation between source–drain current and strain applied to the flexible OFETs make these devices attractive for applications in low-cost strain sensors.

## Results and Discussion

**Figure 1:**
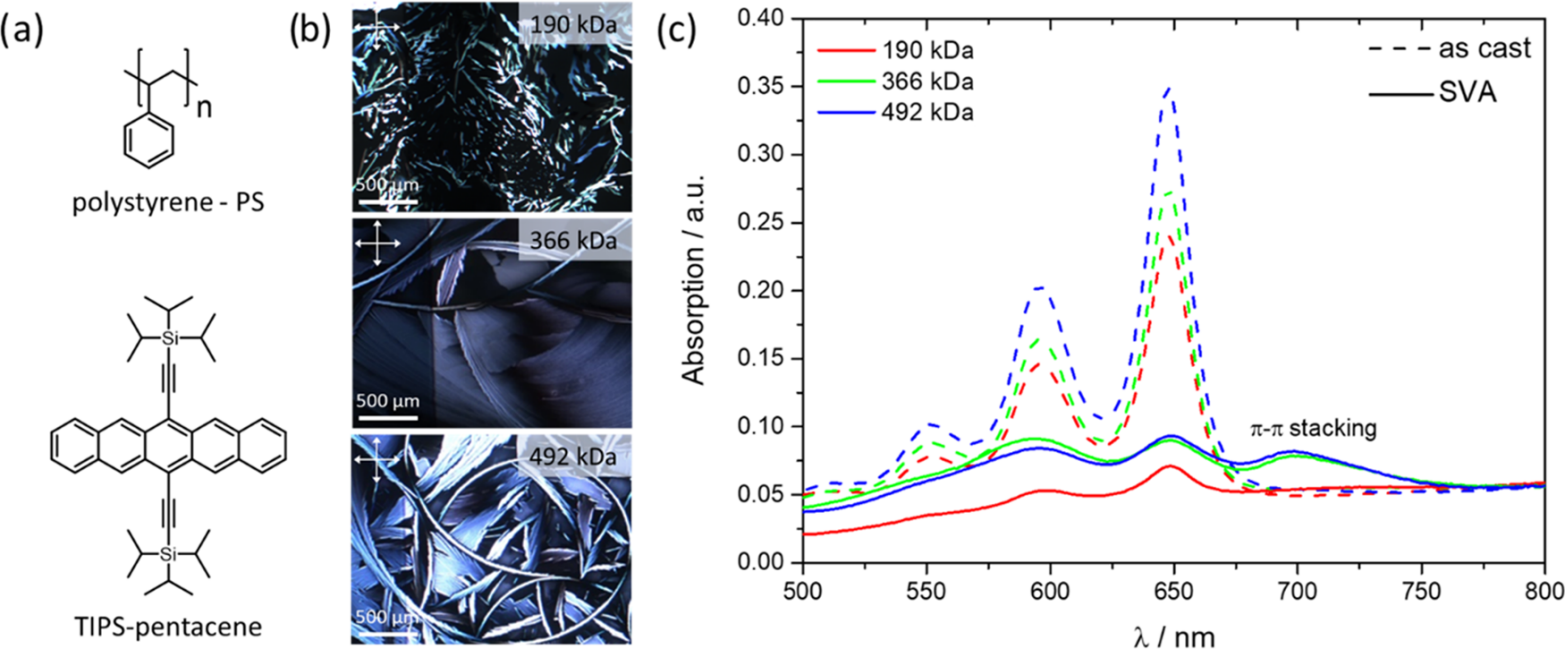
(a) Chemical structures of PS and TIPS-pentacene; (b) polarized optical microscopy images of SVA TIPS-pentacene/PS blend films with different *M*_w_ of PS; and (c) UV–vis spectra of as-cast (dashed lines) and SVA TIPS-pentacene/PS blend films (solid lines) with different *M*_w_ of PS.

TIPS-pentacene was selected as an organic semiconductor for the free-standing films because of its good electrical performance and, more importantly, its intensive molecular π-stacking interactions and strong propensity to form highly crystalline structures, while its high solubility in organic solvents is ensured by silylethynyl groups. As the amorphous insulating polymer with suitable dielectric parameters, PS was applied with *M*_w_ of 190, 366, and 492 kDa to tune the phase separation and blend morphology of the free-standing, self-aligned bilayers (Figure 1a). To fabricate self-aligned films with the substrate serving additionally as a dielectric layer, a higher weight ratio of PS is necessary. Therefore, toluene solution with a TIPS-pentacene/PS ratio of 1:3.3 was first spin cast on rigid auxiliary glass substrates to control the blend morphology during processing. With respect to the literature, where the polymer matrix is mostly used for improvement of the micro- and macrostructure of the organic semiconductor, our work focuses on creating distinct self-standing devices with a spontaneously formed independent dielectric PS layer.^39^ The ratio 1:3.3 was experimentally optimized to create a sufficiently thick dielectric layer to ensure good and stable OFET working parameters, but at the same time, thin enough to induce the flexibility of the devices. As-cast blend films do not show any crystallinity, as evident from black polarized optical microscopy images (POM). Absorption spectra of the corresponding films, presented as dashed lines in Figure 1c, reveal bands that match with the ones for neat TIPS-pentacene in toluene, characteristic for a homogeneous distribution of individual nonstacked semiconductor molecules in the polymer matrix.^49^ Three well-separated intensive distinctive maxima at 550, 595, and 648 nm appear for TIPS-pentacene in the three *M*_w_ of PS. The latter most intense absorption maximum corresponds to the TIPS-pentacene optical band gap of 1.87 eV and is evidence for the lack of π-interactions of the molecules in amorphous films or highly diluted solutions.^50,51^ The intensity of the absorption bands increases for larger film thickness (according to the Beer–Lambert relation) as a consequence of higher solution viscosity with increasing *M*_w_ of polystyrene. As determined by profilometer measurements, the film thickness increases from 700 nm for 190 kDa to 1000 nm for 492 kDa.

**Figure 2:**
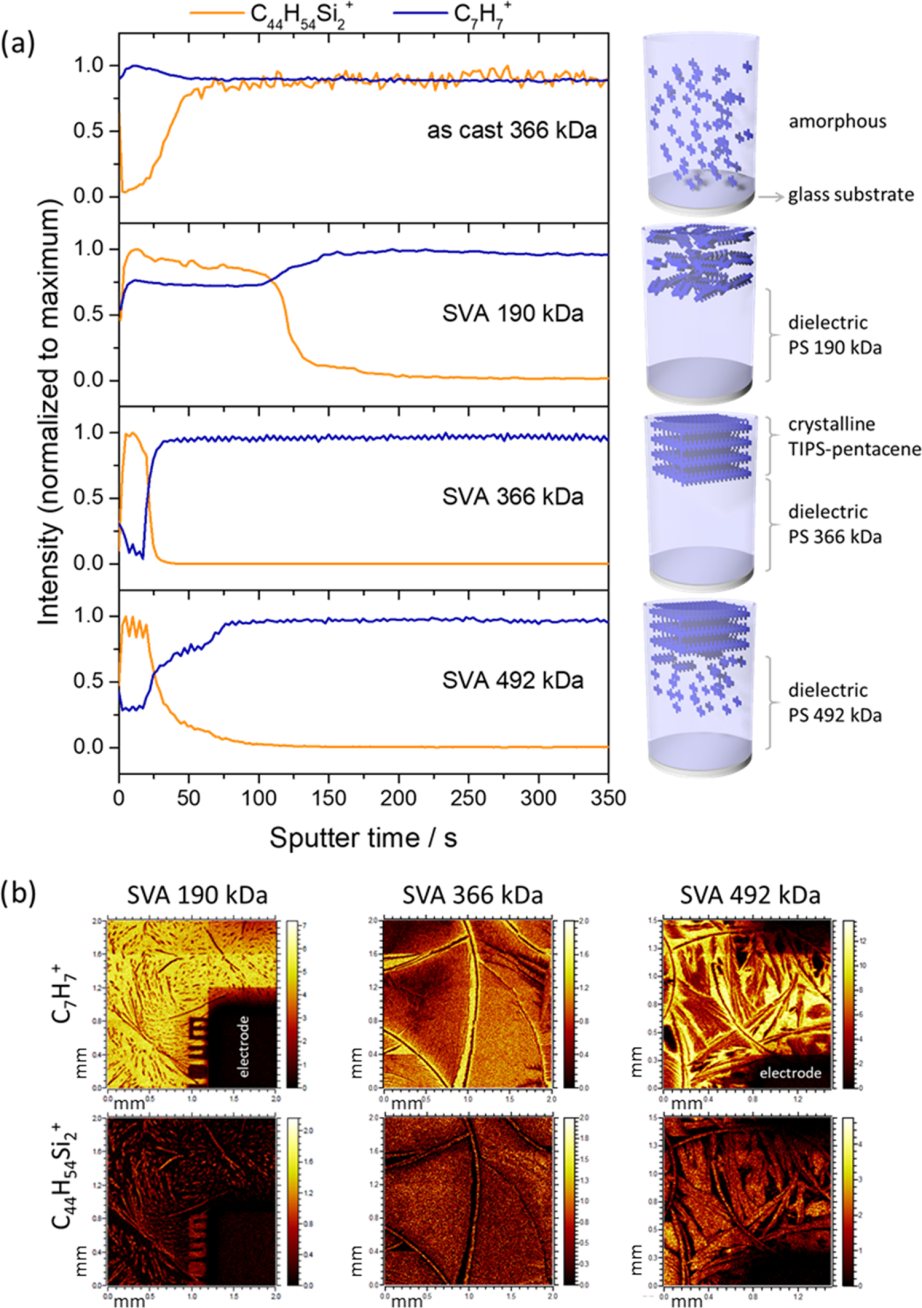
(a) TOF-SIMS depth profiles of as-cast and SVA TIPS-pentacene/PS blend films with schematic illustrations of the phase separation and TIPS-pentacene ordering and (b) lateral distribution of characteristic secondary ion signals at the top film surface of SVA TIPS-pentacene/PS blends obtained by TOF-SIMS imaging analysis (dark areas are OFET electrodes as indicated).

To determine the distribution of TIPS-pentacene in the PS matrix in the as-cast films, time-of-flight secondary ion mass spectrometry (TOF-SIMS) depth profiling was executed. The sputter time indicates the duration of the erosion process and larger times correspond to the composition of deeper layers of the blend film. The detected C_7_H_7_^+^ ions are attributed to PS, while C_44_H_54_Si_2_^+^ ions are assigned to TIPS-pentacene. The depth profiles reveal a plateau region (constant intensity) of both C_44_H_54_Si_2_^+^ and C_7_H_7_^+^ for all as-cast samples with a sputter time of 70 s (Figures 2a and S1a), indicating a homogeneous in-depth distribution of both components through the bulk film. Just at the beginning of the sputtering process, the C_44_H_54_Si_2_^+^ intensity is low, suggesting a lower TIPS-pentacene concentration at the top film surface. The results from POM, UV–vis, and TOF-SIMS demonstrate that in the as-cast blend films, TIPS-pentacene is homogeneously distributed in the polymer matrix and forms a kinetically trapped, thermodynamically metastable state independent of the *M*_w_ of PS.

To induce the thermodynamic order of the organic semiconductor and phase separation between the components into a bilayer structure, the blend films were solvent vapor annealed (SVA). The as-cast films were exposed to toluene vapor in a closed container at 85 °C for 120 min. POM images shown in Figure 1b confirm crystalline TIPS-pentacene in the blend films after SVA, whereby the crystalline morphology strongly depends on the *M*_w_ of PS. While low *M*_w_ results in small needle-like TIPS-pentacene crystals, the intermediate and high *M*_w_ lead to extraordinarily large and homogeneous two-dimensional (2D) domains. Both types of morphologies are common for solution-processed TIPS-pentacene and indicate the underlying crystallization mechanism typical for acenes,^52^ which is related to residual solvent during film casting.^53^ During SVA, solvent molecules penetrate the entire blend film. PS with low *M*_w_ possesses the highest diffusion rate for TIPS-pentacene. This together results in fast crystallization kinetics and short crystalline needles of TIPS-pentacene in a blend film of incomplete phase separation as discussed later in the text (Figure 1b). The higher *M*_w_ of PS decreases the diffusion rate leading to a lower crystallization rate and, in the case of 366 kDa, to a well-defined 2D growth of TIPS-pentacene in large domains (Figure 1b). A further decrease of the kinetics for 492 kDa induces a transition from 2D to 1D fibers formed at high amounts of residual solvent.

The molecular packing of TIPS-pentacene in the SVA blend films was first studied by UV–vis absorption. In comparison to the spectra of the as-cast films, the intensity of the peaks at 510, 550, and 648 nm is strongly reduced, while a new 0–0 transition at 698 nm emerges, indicating the growth of an ordered 2D brickwork TIPS-pentacene phase (solid lines in Figure 1c).^46^ The degree of crystallinity is derived from the ratio of the corresponding intensities at 698 nm for the crystalline phase and at 648 nm for disordered molecules (Figure 1c). From the normalized spectra with subtracted baseline (Figure S2) the 698/648 nm intensity ratio increases from 0.1 for SVA films with 190 kDa PS to 0.7 for 366 kDa PS. These results correspond well to the POM images and confirm the reduced crystallinity of TIPS-pentacene in the blend film with low-*M*_w_ PS. In addition, the low peak intensity at 698 nm related to π-stacking confirms a poor molecular order of TIPS-pentacene in the 190 kDa PS blend. The higher ratio of the peak intensities for 366 kDa indicates higher crystallinity of TIPS-pentacene as observed for the large domains in the POM images. For a higher *M*_w_ of 492 kDa, the peak ratio slightly decreases to 0.65 due to a minor decrease in TIPS-pentacene crystallinity, which is in agreement with the morphology transition from 2D to one-dimensional (1D) structures. The analysis of the UV–vis absorption suggests an optimum *M*_w_ of PS of 366 kDa for the crystallization of TIPS-pentacene in the PS blend.

The crystal structure of TIPS-pentacene in the blend films was further investigated by grazing incidence wide-angle X-ray scattering (GIWAXS). The GIWAXS pattern of the as-cast films exhibits only two broad, amorphous halos at small- and middle-angle ranges (*q* = 0.70 Å^–1^ equal to *d* = 8.97 Å and *q* = 1.36 Å^–1^ equal to *d* = 4.62 Å), both assigned to amorphous PS,^54^ confirming the lack of the molecular order of TIPS-pentacene (Figure S3). After SVA, the blend films reveal high distinct peak intensities that are assigned to the crystalline characteristic form I of TIPS-pentacene.^55^ The molecules exhibit slipped π-stacking with a neighboring distance of 3.3 Å. In the crystalline, phase-separated layer, the stacking direction is oriented in the plane parallel to the substrate surface with edge-on arranged molecules as in the case of vacuum-sublimed and solution-processed TIPS-pentacene films (Figure S3).^56^ This molecular organization is considered to be beneficial for the charge carrier transport in OFETs. For the operation of OFETs, an individual TIPS-pentacene phase is additionally required for the percolation pathways of charge carriers.

The TOF-SIMS depth profiles provide information about the vertical phase separation between TIPS-pentacene and PS in the as-cast and SVA blend films (Figure 2a). After SVA, the blend morphology significantly changes in comparison to as-cast films described earlier in the text. The bulk films consist of neat PS, while the homogeneity of the TIPS-pentacene top layer depends on the *M*_w_ of the polymer. For *M*_w_ of 190 and 492 kDa, the TIPS-pentacene top layer is intermixed with PS so that the interface between both fractions becomes undefined. In the case of 366 kDa, the TIPS-pentacene-rich top phase contains only a minor amount of PS, while the interface between both major phases is sharp and distinct. The mechanism for bilayer formation is related to solute–substrate interactions. During SVA, PS is swollen by the solvent molecules, increasing the diffusion of the TIPS-pentacene molecules within the polymer matrix. PS with its higher affinity to the hydrophilic surface preferentially accumulates at the interface with the auxiliary glass substrate, while TIPS-pentacene of lower surface energy migrates to the air/film interface as the top layer. According to the literature, the average water contact angle for the TIPS-pentacene layer is 100.9°,^57^ whereas the average water contact angle for the PS layer is 94°.^58^ This proves that despite the hydrophobic character of PS, it still has a higher affinity to the hydrophilic surface than TIPS-pentacene; thanks to this, PS accumulates at the bottom of the blend film during SVA. The differences in the homogeneity of the TIPS-pentacene top layer are related to the *M*_w_ and thickness of the PS fraction, leading to different diffusion times of the semiconducting molecules out of the PS matrix during SVA. In the blend with 190 kDa PS, TIPS-pentacene does not completely phase-separate out of the polymer matrix and partly remains in the kinetically trapped state (see illustration in Figure 2a). A similar blend morphology is observed for blends with 492 kDa PS due to large film thickness and low TIPS-pentacene diffusion. These observations are in line with the results obtained for PαMS^59^ and PEO^60^ where diffusivity and crystallization of the organic semiconductor are reduced for high-*M*_w_ polymers. The optimum conditions are found for 366 kDa at which TIPS-pentacene almost completely phase-separates with PS and forms large and highly crystalline domains in the top layer.

To determine the actual layer thickness of TIPS-pentacene in the bilayer with PS of 366 kDa, a depth-corrected SPM profile across the sputter crater was performed (Figure S1b). The scan is acquired for the interfaces of TIPS-pentacene/PS and PS/substrate. The measured depth values of the interfaces are used to convert the sputter time to a depth scale. A thickness of 150 nm for the TIPS-pentacene top layer is determined at a complete bilayer thickness of 850 nm, which is in agreement with the profilometer results. This bilayer structure corresponds well to a typical transistor configuration with an active semiconducting layer on a dielectric and substrate.

TOF-SIMS surface imaging was performed to gain a more detailed insight into the phase homogeneity of the TIPS-pentacene layer located on top of the SVA blend films (Figure 2b). The signal distribution of the ion intensity confirms the results from the depth profiles (Figure 2a). The blend films with PS of 190 and 492 kDa show both C_44_H_54_Si_2_^+^ and C_7_H_7_^+^ signals, confirming an intermixed phase of TIPS-pentacene and PS at the top surface. Thereby, the displayed morphologies correspond well to the POM images shown in Figure 1b. In the TOF-SIMS surface images shown in Figure 2b, independently from the *M*_w_ of PS, the crystalline TIPS-pentacene structures reveal distinct C_44_H_54_Si_2_^+^ signals, while their surrounding leads to pronounced C_7_H_7_^+^ intensity. These chemical maps indicate that the crystalline TIPS-pentacene structures after SVA are embedded in the PS matrix at the top surface. In the case of PS of 190 and 492 kDa, phase separation occurs during SVA, but the TIPS-pentacene crystals do not completely penetrate out of the PS phase. The surface morphology differs for blend films with PS of 366 kDa. The observed C_7_H_7_^+^ intensity is lower for SVA blend films over the entire surface area, while the C_44_H_54_Si_2_^+^ signal remains on a similar level (Figure 2b). High PS intensity is only observed for domain boundaries that appear birefringent in the POM images. This surface analysis agrees with the depth profile shown in Figure 2a that exhibits for the top surface high intensity mainly for TIPS-pentacene. These results indicate that for blend films with PS of 366 kDa predominantly, a rich TIPS-pentacene phase containing only a minor PS fraction on top of the pure PS bulk film is formed. Atomic force microscopy (AFM) images shown in Figure S4 display elongated and 5 μm wide TIPS-pentacene crystals on top of the PS film (366 kDa). A crystal height of approximately 100 nm corresponds to the depth-corrected SPM profile and profilometer results.

**Figure 3:**
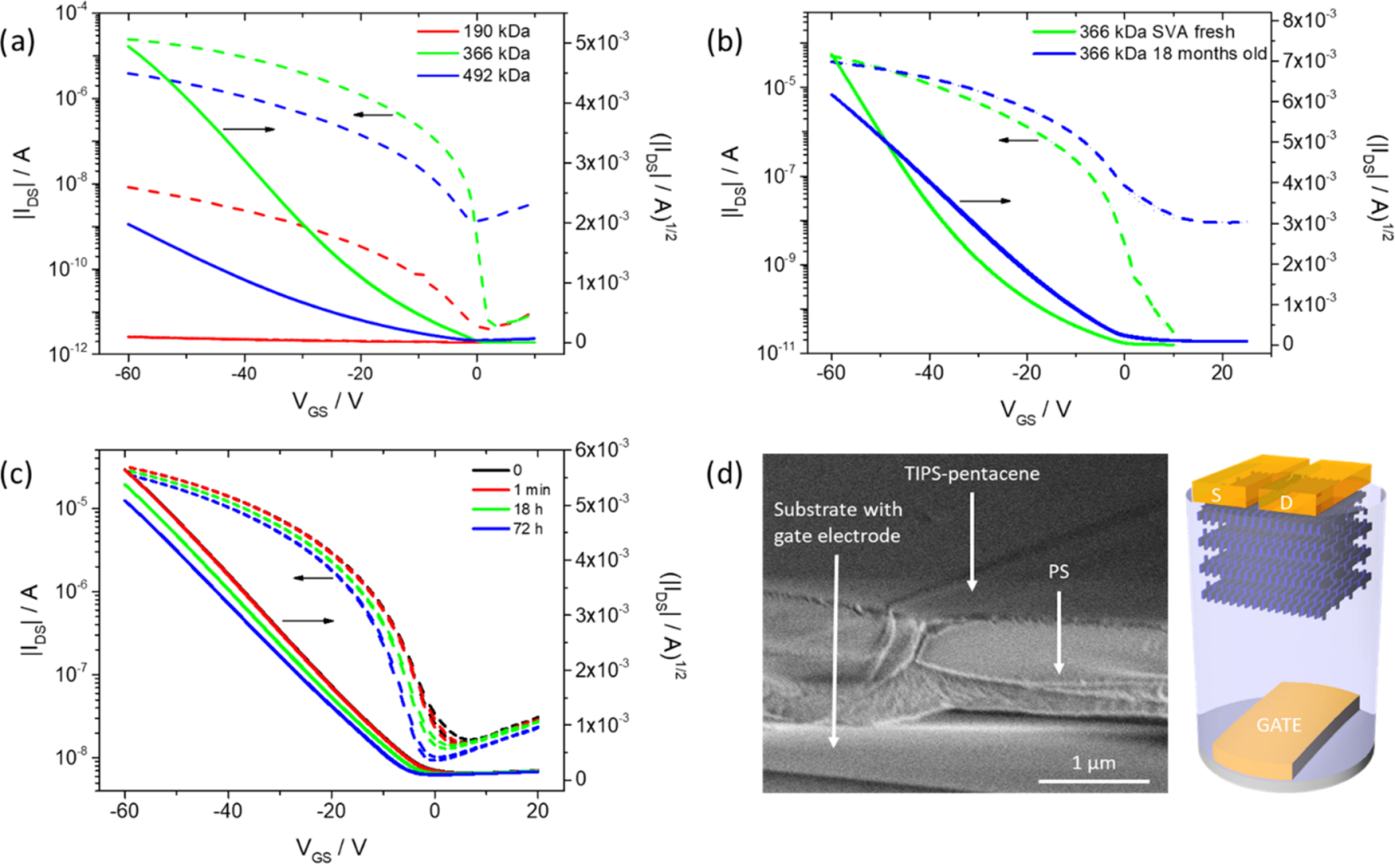
(a) Transfer characteristics at *V*_DS_ = −60 V of OFETs based on SVA TIPS-pentacene/PS blend films with different *M*_w_ of PS; (b) transfer characteristics at *V*_DS_ = −60 V of OFETs based on SVA blends of TIPS-pentacene/366 kDa PS fresh (green) and after 18 months storage in air (blue); (c) transfer characteristics after different stressing times of OFETs based on SVA blends of TIPS-pentacene/366 kDa PS; and (d) cross-sectional scanning electron microscopy (SEM) image of the SVA TIPS-pentacene/366 kDa PS blend and the schematic illustration of the OFET architecture.

The charge carrier transport of the blend films was studied in top contact, bottom gate OFETs (Figure 3d). The SVA blend films were deposited on auxiliary glass substrates with an evaporated gate electrode. The source and drain electrodes were evaporated after the SVA process. As-cast films do not reveal any field effect due to the homogeneous molecular distribution of TIPS-pentacene in the PS matrix. After SVA, all bilayers show a p-type charge carrier transport with low turn-on voltage *V*_ON_, whereby the mobility strongly depends on the blend morphology determined by the *M*_w_ of PS (Figure 3a and Table 1). For the poorly crystalline small TIPS-pentacene needles formed in the blend with PS of 190 kDa, a charge carrier mobility (μ_MAX_) of only 1 × 10^–4^ cm^2^ V^–1^ s^–1^ is determined (average charge carrier mobility of 8 × 10^–5^ cm^2^ V^–1^ s^–1^). This value increases to 4 × 10^–1^ cm^2^ V^–1^ s^–1^ for large 2D TIPS-pentacene domains in blends with PS of 366 kDa (average charge carrier mobility of 2 × 10^–1^ cm^2^ V^–1^ s^–1^), including a high on/off ratio of 6 × 10^6^. The maximum charge carrier mobility found for the blend with PS of 366 kDa is on a similar level as for TIPS-pentacene rigid devices reported in the literature.^52,61^ In summary, the difference in the charge carrier transport is caused by the TIPS-pentacene crystal size that is influenced by different phase separation with PS with various molecular weights. Charge carrier traps located at crystal boundaries between small TIPS-pentacene needles in 190 kDa PS significantly reduce the charge carrier transport in comparison to large crystals observed in PS of 366 and 492 kDa. With respect to 366 kDa, the device performance slightly drops to 1 × 10^–1^ cm^2^ V^–1^ s^–1^ for 492 kDa (average mobility of 8 × 10^–2^ cm^2^ V^–1^ s^–1^) due to the incomplete vertical phase separation. The subthreshold swing also correlates with the TIPS-pentacene morphology in the blend films with values of 7.7 V/dec for 190 kDa, 1.0 V/dec for 366 kDa, and 6.2 V/dec for 492 kDa. In all cases, a gate leakage of only 9.9 × 10^–8^ A (at *V*_GS_ = *V*_DS_ = −60 V) is recorded, which is 2 orders of magnitude lower than *I*_DS_, confirming a complete and defect-free coverage of the gate electrode by the phase-separated PS layer. Figure S5 presents transfer characteristics with *I*_GS_ and *I*_DS_ for the TIPS-pentacene/366 kDa PS bilayer.

**Table 1:** OFET Parameters for SVA TIPS-Pentacene/PS Blend Films with Different *M*_w_ of PS

PS *M*_w_ [kDa]	μ_MAX_ [cm^2^ V^–1^ s^–1^]	*V*_ON_ [v]	*V*_Th_ [V]	ON/OFF ratio
190	1 × 10^–4^	0	–15	2 × 10^3^
366	4 × 10^–1^	2	–10	6 × 10^6^
492	1 × 10^–1^	–1	–15	3 × 10^3^

Organic semiconductors typically suffer from low environmental stability since humidity or oxygen can trap charge carriers reducing the device performance. To evaluate the long-term environmental stability of OFETs based on the SVA TIPS-pentacene/366 kDa PS blends, the devices were exposed to air for 18 months under typical ambient conditions. The aged devices reveal 3 orders of magnitude increased OFF current, but still on a low and acceptable level (Figure 3b). On the other hand, the aged devices exhibit close-to-ideal linear transfer characteristics with a negligible threshold voltage (*V*_Th_) close to 0 V, and the reliability factor improved from 40% for fresh OFETs to 86%. The increase of the OFF current and shift of the threshold voltage can be explained by the oxygen doping of the semiconductor film, which induced charges in the TIPS-pentacene film that cannot be depleted by the gate voltage.^62^ As a further effect for the device operation, moderate doping of P3HT/PS blends by oxygen increases the device performance over time as reported for P3HT/PS blends.^63^ An identical effect due to oxygen doping probably also occurs for the self-standing TIPS-pentacene/PS bilayer OFETs, improving additionally the operational stability together with reliability.

The operational stability under bias stress is another important aspect for the applicability of OFETs.^64^ It was recently reported that the bias stress stability of TIPS-pentacene is improved by blending with PS. The decrease in drain current after 2 h of stressing was reduced to 30% for a TIPS-pentacene/PS blend compared to 80% for neat TIPS-pentacene OFETs.^65^ In our study, OFETs based on free-standing SVA TIPS-pentacene/366 kDa PS blends were stressed by applying continuous gate and drain biases of −60 V for 72 h, which was interrupted for recording the transfer characteristics (Figure S6a,c). As evident from the transfer curves shown in Figure 3c, *V*_Th_ is only shifted from −2.2 to −6.1 V after 72 h. This shift corresponds to a decrease of the maximum saturation current to 0.7 × 10^–5^ A in the output curves and a reduction of only 18% toward the initial value (Figure S6b), confirming the pronounced operational stability of the devices. The electrical stability can be explained by the trap-free dielectric semiconductor interface in the phase-separated bilayer blend. In addition, the trap-free interface is confirmed by the lack of hysteresis in the transfer characteristics (Figures 3 and S11) and in stable drain current during the bias stress experiment (Figure S6). The SEM image shown in Figure 3d (and Figure S12) confirms the complete vertical phase separation that was concluded from the TOF-SIMS study and exhibits a smooth semiconductor/dielectric interface required and undisturbed transport of charge carriers.^66^

To our best knowledge, the obtained charge carrier mobility of 0.4 cm^2^ V^–1^ s^–1^ is one of the highest in comparison to other spin-coated devices.^67^ In addition (see Figure S6), a negligible bias stress effect is observed when it is compared to the results presented above. We can conclude that our deposition technique allows us to fabricate a device that can be compared to the “ultraflexible solution-processed organic field-effect transistors” with a mobility of 0.2 cm^2^ V^–1^ s^–1^ with the proviso that our dielectric and semiconductor films are deposited from single solution.^67^

**Figure 4:**
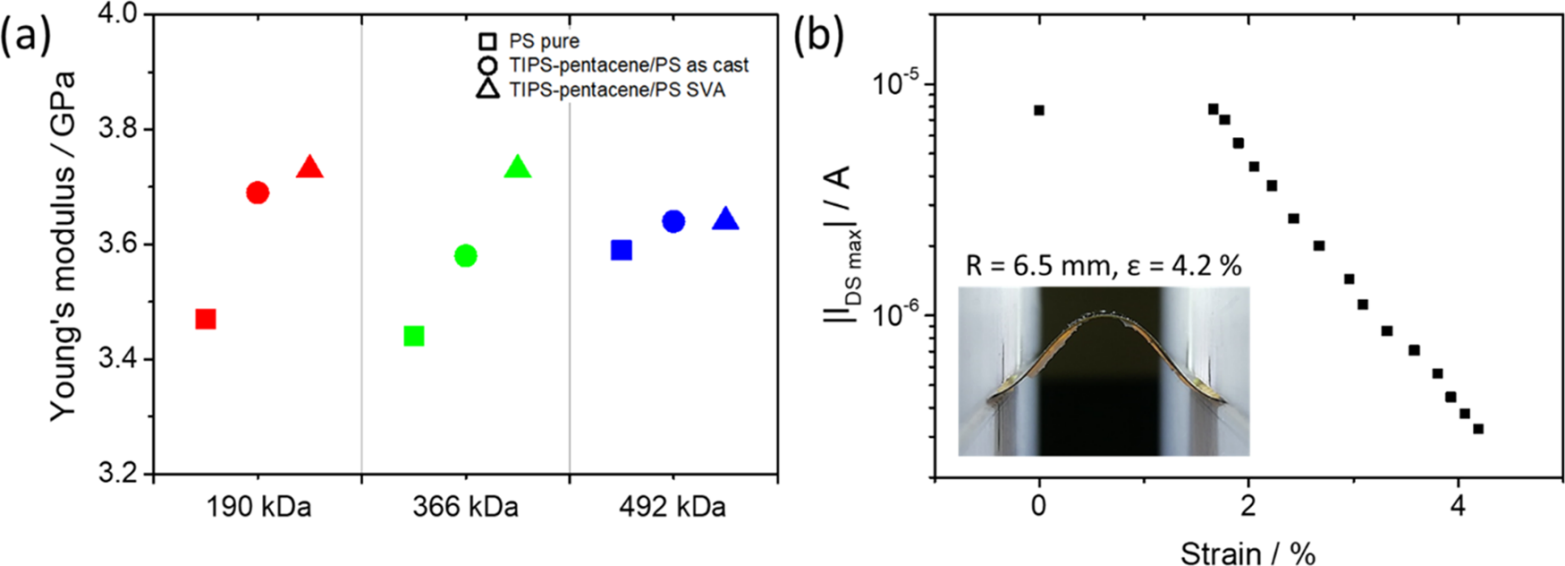
(a) Young’s modulus for PS (square), as-cast (circle), and SVA (triangle) TIPS-pentacene/PS blend films with 190 kDa (red), 366 kDa (green), and 492 kDa (blue) PS, (b) *I*_DS_ as a function of the strain of an OFET based on the SVA TIPS-pentacene/PS blend. The inset shows the bending of the SVA TIPS-pentacene/PS OFET by applying convex buckling through compression (*R*, radius and ε, strain).

As mentioned in the Introduction section, mechanical deformation of OFETs is desired for future flexible electronics. For this reason, two experiments describing the mechanical properties of the TIPS-pentacene/PS bilayers were performed. In the first study, the intrinsic elasticity of the films before and after the phase separation was evaluated by employing Brillouin light scattering (BLS).^68−70^ This method probes velocities of thermally excited acoustic waves, typically of GHz frequencies and submicrometer wavelengths. It allows for contactless and nondestructive evaluation of the elastic properties of transparent and opaque materials. The TIPS-pentacene/PS films can be considered a slow-on-fast system, as the relatively soft polymer film is placed on a stiff auxiliary glass substrate. In this case, the probed acoustic waves are strongly localized in the film (Figure S9d).^69^ The BLS measurements of the reference neat PS films reveal Young’s modulus (*E*) similar to the one known from the literature (Figure 4a). The variation of the determined values is small and can be explained by the different molecular weights of PS. For the as-cast TIPS-pentacene/PS blend films, we found that *E* increases with respect to pristine PS films. This effect is more pronounced (6–7%) for the films consisting of lower-*M*_w_ PS. Based on Wood’s law^70^ and data obtained for 366 kDa PS, the stiffening of the film results from the addition of TIPS-pentacene having an effective Young’s modulus of about 7 GPa. These results clearly confirm that homogeneously distributed TIPS-pentacene molecules do not significantly change the Young’s modulus of the as-cast TIPS-pentacene/PS films. The phase separation leads to a slight increase of the effective Young’s modulus but it is still much lower than that for pure TIPS-pentacene. This change (about 4%) is most obvious for the bilayer film with 366 kDa PS. This observation correlates well with the distinct phase separation and high crystallinity of TIPS-pentacene in the SVA blend film. Overall, the observed differences between pristine PS and amorphous and phase-separated TIPS-pentacene/PS samples can be monitored by BLS. However, the variation is relatively small, and it can be concluded that the films maintain their elastic properties and potential flexibility upon phase separation, which are mainly determined by the Young’s modulus of PS compared to TIPS-pentacene, and that the PS matrix significantly improves the intrinsic elastic properties of TIPS-pentacene/PS films.

To prove the concept of flexible and working OFETs based on free-standing bilayer films, the influence of bending strain on the charge carrier transport was investigated for the SVA blend of TIPS-pentacene/492 kDa PS. The free-standing bilayer was deposited on a 135 μm thick Kapton carrier foil equipped with the gate electrode. The thick carrier foil was necessary to facilitate ease of handling of the bilayer OFET and to increase the bending strain of the sample. The source and the drain electrodes were evaporated after SVA. The semiconductor deposition, device handling, and mechanical bending were performed in ambient conditions at room temperature. Before the bending experiments were executed on the OFETs, the bending behavior of neat spin-cast PS films was studied. The neat PS films were bent in an analogous manner to the OFET samples, and morphological changes were analyzed at each strain by optical microscopy. At strains below 4%, the PS film remains intact without any damage. However, for strains above 4%, long wrinkles appear in the films after returning to the flat position (Figure S7a). The density of the wrinkles increases with higher strain. The wrinkles are related to elongation of the PS film beyond the yield point of 4% at which permanent deformation of the material occurs.^71^

After characterization of the OFETs in the planar geometry, the device characteristics were monitored during gradual bending. To obtain high precision of the strain on the sample, the film was compressed between two holding clamps to induce convex buckling (Figures 4a (inset) and S8). At each position, a calibrated microscopy image of the sample was taken to determine the bending radius of the transistor channel. In addition to the transfer and output characteristics of the OFETs, the gate leakage was recorded for each bending radius to ensure the reliability of the PS dielectric layer. The gate leakage was on a very low level of only 10^–9^ A for all bending radii. Figure 4b shows the maximum *I*_DS_ as a function of strain in the range of 0–4.2%. The *I*_DS_ current remains constant to a strain of 1.8%, indicating mechanical stability of the OFETs at the corresponding deformation. This is slightly higher stability than that reported in the literature for TIPS-pentacene drop cast on the Mylar foil, which revealed constant operation until 1% strain.^67^ The improvement in the mechanical stability of the OFETs is attributed to the PS dielectric of lower Young’s modulus in the bilayer blend film, absorbing part of the stresses during bending. Above a strain of 1.8%, *I*_DS_ gradually decreases following a close logarithmic relationship to the strain. The decrease of current requires further investigation; however, one of the possible explanations is related to the permanent plastic deformation of the PS dielectric layer beyond its yield point, leading to drifting and separation of the TIPS-pentacene crystalline domains and subsequent breakage of the percolation paths. As the transfer characteristics, shown in Figure S7b, measured before and after bending show no shift, the effect of the bias stress can be neglected.^64^ However, the decrease in *I*_DS_ may be related to defects such as wrinkles, cracks, or delamination during deformation of the bilayer film. The close relation between strain and current makes these devices attractive for applications in low-cost strain sensors.

## Conclusions

Free-standing, self-aligned, and flexible TIPS-pentacene/PS bilayer films were fabricated for OFETs applications. To induce crystallization of TIPS-pentacene into large domains in the blend with PS, SVA was applied and the *M*_w_ of the polymer was optimized. Under optimized conditions, a phase-separated bilayer structure was formed, with a homogeneous TIPS-pentacene top active layer and PS serving as a dielectric and substrate. PS provides an excellent combination of required properties for the fabrication of self-standing reliable bilayer OFETs. PS with the right *M*_w_ ensures distinct phase separation with TIPS-pentacene into the desired bilayer morphology due to the right Δ*G*_m_ and χ in the blend. As described, the distinct phase separation leads to a well-defined interface between both phases that is responsible for the exceptional operational stability of the devices. Furthermore, the mechanical strength of PS enables facile handling by a defect-free peeling off the bilayer film from the rigid substrate. The corresponding OFETs showed charge carrier mobilities similar to rigid devices and pronounced environmental and bias stress stabilities that were attributed to a well-defined and trap-free interface between the organic semiconductor and the dielectric polymer. Finally, Young’s modulus and elongation of yield allow the construction of flexible bilayer OFETs that operate without reduction in performance up to a strain of 2% that is a remarkable value for crystalline solution-processed molecular semiconductors.

## Experimental Section

### Materials

6,13-Bis(triisopropylsilylethynyl)-pentacene (TIPS-pentacene) was purchased from Ossila Ltd. Polystyrene with different molecular weights was synthesized at Max Planck Institute for Polymer Research in Mainz, Germany.

Four PS masses were chosen for our studies: 35, 190, 366, and 492 kDa. During the bilayer fabrication, the blend with PS of 35 kDa does not create a homogeneous and continuous layer and cannot be separated from the rigid substrate. The corresponding OFETs with 35 kDa show an unreliable performance in comparison to other blends (Figure S10b). For this reason, PS with *M*_w_ lower than 190 kDa is not suitable for the self-standing bilayers applied in OFETs.

The weight average molecular weights (*M*_w_) were 190, 366, and 492 kDa the number average molecular weights (*M*_N_) were 173, 338, and 444 kDa; and polydispersity (PDI) values were 1.09, 1.08, and 1.11, respectively (Table 2). Toluene HPLC grade was purchased from Sigma-Aldrich. Ultraflat glass substrates coated with a 20 nm layer of synthetic quartz were purchased from Ossila Ltd. All compounds and solvents were used as received.

**Table 2:** Parameters of Used PS with Three Different *M*_w_

PS [kDa]	number average	weight average	peak mol W.	dispersity
190	173 304	189 045	189 497	1.09
366	337 674	365 577	372 787	1.08
492	444 427	492 092	519 514	1.11

### Blend Solution Preparation

TIPS-pentacene solution (15 mg mL^–1^) was mixed with PS solution (60 mg mL^–1^) in a ratio of 1:3.3. Toluene was used as a solvent for both solutions.

### Sample Preparation

First, glass substrates were cleaned in an ultrasonic bath of acetone and isopropanol two times for 15 min each. Blend solution was spin cast (60s, 750 rpm) on substrates with an evaporated 90 nm thick gold gate electrode. Afterward, solvent vapor annealing (SVA) of the blend film was performed using toluene vapor at 85 °C. Samples were closed in a sealed container with the solvent on a hot plate for 6 h. A temperature of 85 °C increased the vapor pressure (from 3 kPa at room temperature to 46 kPa at 85 °C)^72^ and allowed the vapor to penetrate into deeper layers of the film,^44^ which increased the mobility of the polymer and semiconductor molecules and intensified the phase separation.

Gold source and drain electrodes of 50 nm thickness were thermally evaporated through shadow masks. Samples with different molecular weights of PS were prepared in the same way. Samples for the bending tests were prepared on a 135 μm thick Kapton foil instead of auxiliary glass substrates. The foils were cut to size, then washed with water and isopropanol, and wiped with a lint-free cloth. Next, the 90 nm thick gold gate electrode was deposited by thermal evaporation. On the foil substrate blend solution was spin-cast in the same conditions like for samples on glass substrates. Afterward, SVA of the blend film was performed using toluene vapor at 85 °C. At the end, special gold source and drain electrodes of 50 nm thickness were thermally evaporated through shadow masks.

### Techniques

#### Polarized Optical Microscopy (POM) and Optical Microscopy (OM)

The morphology of the blend films was investigated using a Zeiss microscope (with polarizing filters) equipped with a Hitachi KP-D50 color digital CCD camera and a Keyence VHX-7000 (with polarizing filters).

#### UV–vis Spectroscopy

Absorption spectra of the blend films were measured using a PerkinElmer Ltd spectrometer 900R with an all-reflecting, double-monochromator optical system with holographic gratings used in each monochromator for the UV–vis range. Spectra were collected in the range of 500–800 nm.

#### Grazing Incidence Wide-Angle X-ray Scattering (GIWAXS)

GIWAXS measurements of blend films were performed at the TU Dortmund DELTA Synchrotron. The beam size was 1.0 × 0.2 mm^2^ (width × height), and the samples were irradiated just below the critical angle for total reflection with respect to the incoming X-ray beam (∼0.1°). The scattering intensity was detected on a two-dimensional image plate (MAR-345) with a pixel size of 150 μm (2300 × 2300 pixels), and the detector was placed 523 mm from the sample center. All X-ray scattering measurements were performed under vacuum (∼1 mbar) to reduce air scattering and beam damage to the sample. All data processing and analysis were performed using the software package Data squeeze.

#### Time-of-Flight Secondary Ion Mass Spectrometry (TOF-SIMS)

Depth profiles, surface images, and SPM profiles of the blend films were acquired using an IONTOF TOF.SIMS^5^ NCS. Surface images were collected at a cycle time of 150 μs (mass range: 1–2070 μ), using 30 keV Bi_3_ primary ions at a current of 0.11 pA. Depth profiles were acquired at identical analysis conditions, employing an analysis area of 200 × 200 μm^2^ and a gas cluster sputter gun using 5 keV cluster ions (Ar_1500_) at a current of 2.5 nA with a sputter area of 400 × 400 μm^2^. SPM profiles were collected with the use of a contact-mode cantilever PPP-CONTSC, 0.2 N m^–1^, with setpoint parameters 12 nm, *P* = 0.3 nm nm^–1^, *T*_i_ = 0.5 ms, a segment overlap of 10 μm, and a scan length of ∼800 μm with a velocity of 4000 nm s^–1^.

#### Atomic Force Microscopy (AFM)

Morphology inspections were conducted with the use of dimension icon microscopy in the tapping mode.

#### Organic Field-Effect Transistors

Each sample consisted of 20 OFETs in the bottom gate, top contact configuration with channel lengths from 10 to 80 μm and a width of 1 mm. Electrical measurements were conducted in a glovebox filled with nitrogen with reduced humidity. Transistor characterization was performed using a Keithley 2634B source meter connected to a needle-probe station, under a drain bias of −60 V and gate biases varied from 10 to −60 V.

For charge carrier mobility calculation, ε = 2.6 was used as the dielectric constant of PS; in addition, approximations of dielectric thickness were performed. The average thickness of the whole system was used as the thickness of the dielectric. Mobility was extracted from the liner region of transfer characteristics in the range from −30 to −60 V.

At least 15 working transistors with various channel lengths from each PS mass were characterized. For the blend with PS of 190 and 492 kDa, only 20% of OFETs showed the field effect on the sample, and the number of working devices increased and reached 50% for the blend with PS of 366 kDa. Mainly, OFETs from the edges of the samples did not work due to the “edge effects” like poor organization, poor separation, and larger thickness in comparison to the center of the sample, induced by spin coating and high viscosity of solutions.

#### Brillouin Light Scattering (BLS)

To determine the Young’s modulus *E* of the films, Brillouin light scattering was employed in the VV (vertical polarization of the incident and scattered light) transmission geometry configuration. Measurements were performed at room temperature using a six-pass tandem Fabry-Perot interferometer and a 532 nm CW laser as the incident light source. More detailed description of this method is provided in the Supporting Information (Figure S9).

#### Bending Tests

The transistor samples were prepared on a Kapton foil, which improved the handling and increased the applied strain on the semiconducting layer. To ensure a low signal-to-noise ratio of the measurement, several steps were taken to isolate each of the transistor signals and ensure stable contact of the electrodes. Each sample consisted of five OFETs in the bottom gate, top contact configuration with a channel length of 30 μm and a width of 1 mm. Electrical measurements were conducted in open air and room-temperature conditions. Transistor characterization was performed using a Keithley 2634B source meter connected to the bending setup, under a drain bias of −60 V and gate biases varied from 10 to −60 V. More information about the bending procedure is provided in the Supporting Information (Figure S8).

## Supporting Information Available

Additional experimental details, including TOF-SIMS data processing (Figure S1); results of UV–vis, GIWAXS, AFM characterization, and electrical characterization (Figures S2–S7 and S10–S12); and detailed parameters of the bending test and the BLS experiment (Figures S8, S9 and Table S1) (PDF)
