# Supplementary material for: Self-Aligned Bilayers for Flexible Free-Standing Organic Field-Effect Transistors
Source: ACS Appl Mater Interfaces. 2021 Dec 4;13(49):59012–22. doi: 10.1021/acsami.1c15208 (PMC8678985; doi:10.1021/acsami.1c15208)
Supplement: Supplementary file 1 — am1c15208_si_001.pdf [file am1c15208_si_001.pdf]

Supporting Information

Self-aligned bilayers for flexible free-standing organic field-effect transistors

Hanna Zajaczkowska,[1] Lothar Veith,[2] Witold Waliszewski,[1] Malgorzata A. Bartkiewicz,[2,3] Michal Borkowski,[1] Piotr Sleczkowski,[1] Jacek Ulanski,[1] Bartlomiej Graczykowski,[2,3] Paul W.M. Blom,[2] Wojciech Pisula,[1,2,*] Tomasz Marszalek[1,2,*]

[1] Department of Molecular Physics, Faculty of Chemistry, Lodz University of Technology, Zeromskiego 116, 90-924 Lodz, Poland
[2] Max Planck Institute for Polymer Research, Ackermannweg 10, 55128 Mainz, Germany
[3] Faculty of Physics, Adam Mickiewicz University, Uniwersytetu Poznanskiego 2, Poznan 61-614, Poland

*Correspondence: pisula@mpip-mainz.mpg.de, marszalek@mpip-mainz.mpg.de

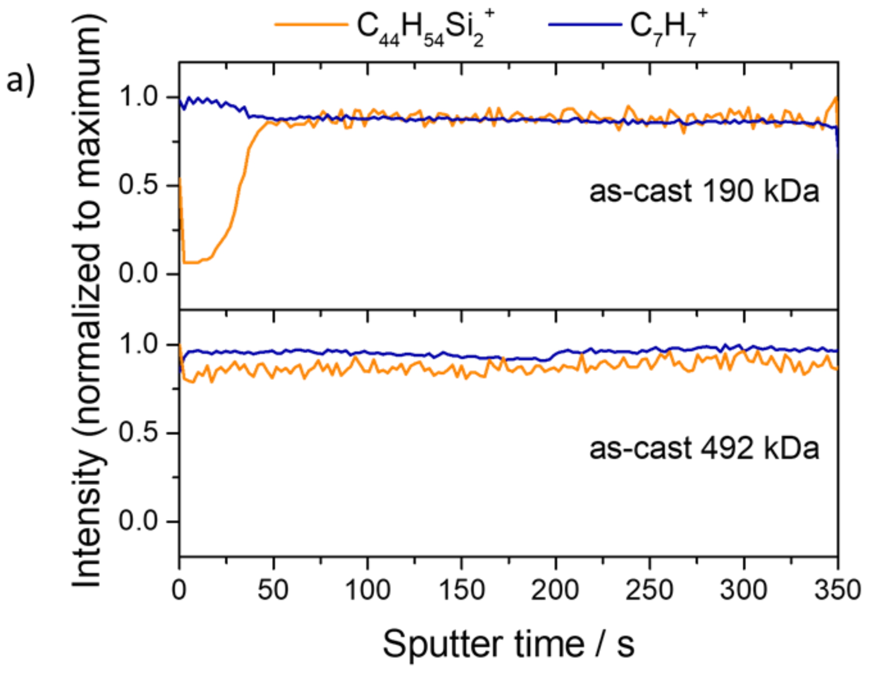

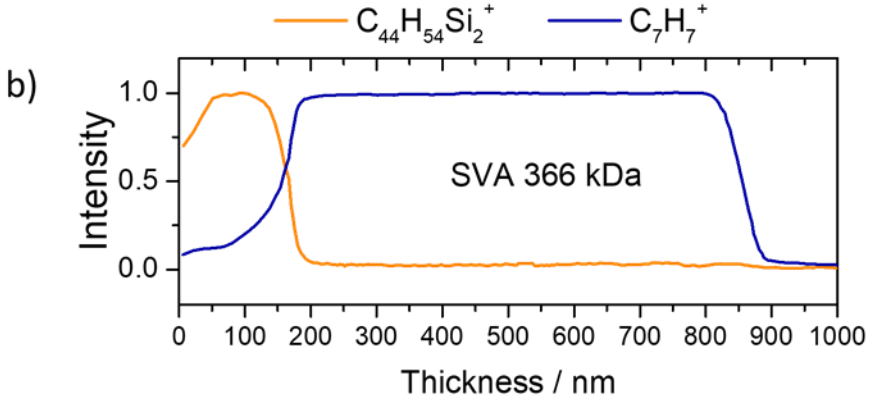

Figure S1. a) TOF-SIMS depth-profiles of as cast TIPS-pentacene/PS blend films for PS of 190 kDa and 492 kDa, b) TOF-SIMS depth-profile of SVA TIPS-pentacene/PS 366 kDa blend film as a function of film thickness.

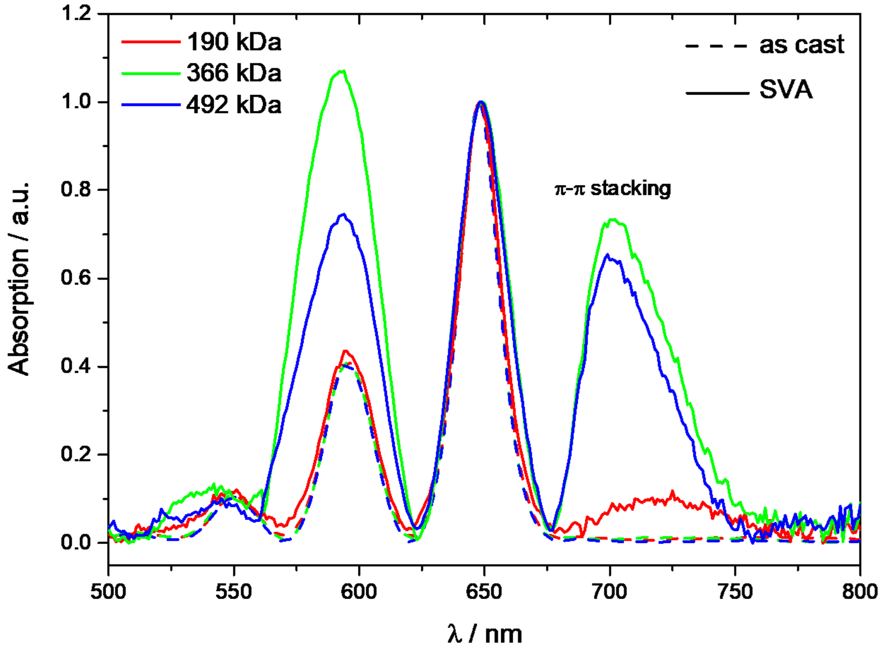

Figure S2. UV-VIS normalized spectra with subtracted baseline of as cast and SVA TIPS-pentacene/PS blends with different M_W of PS.

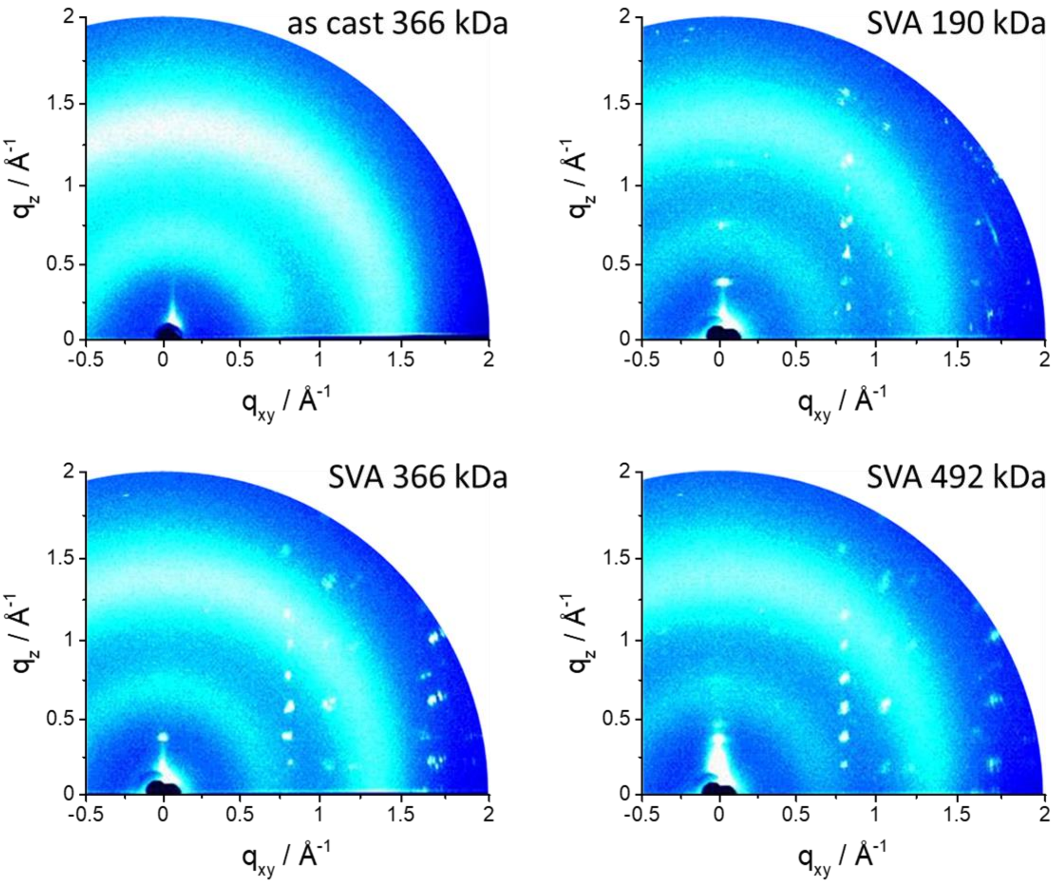

Figure S3. GIWAXS patterns of as cast and SVA TIPS-pentacene/PS blends with PS of different molecular weight.

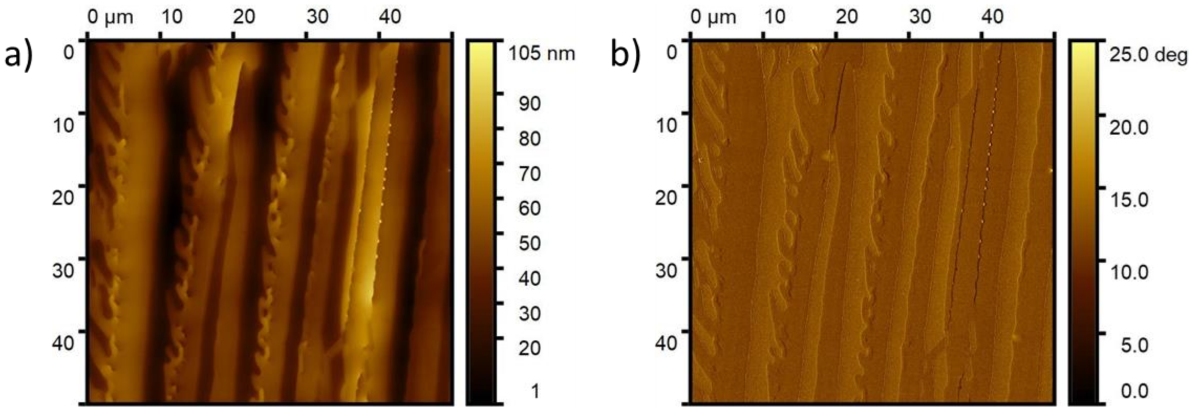

Figure S4. a) Height and b) phase AFM images for as cast blends of TIPS-Pentacene/366 kDa PS after solvent vapor annealing

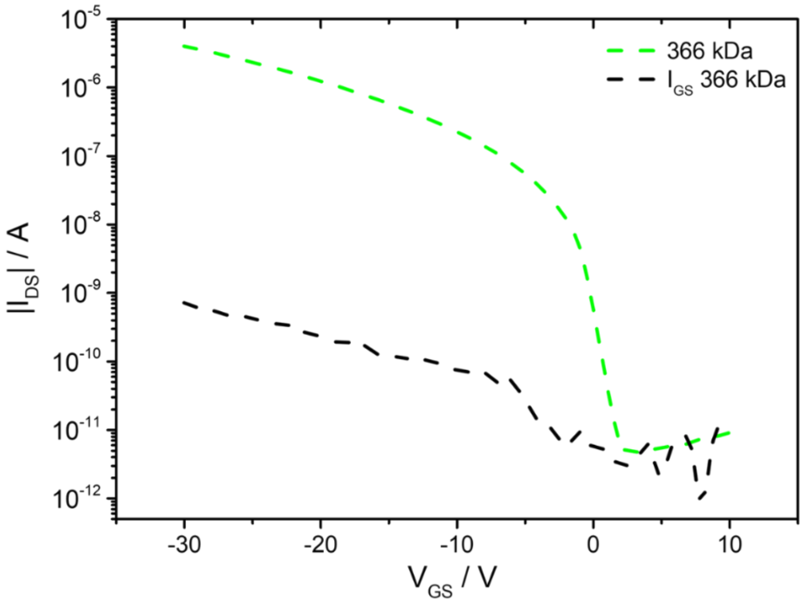

Figure S5. Transfer characteristics at V_{DS}= - 60 V of OFET based on SVA blend of TIPS-pentacene/366 kDa PS with corresponding I_{GS} for the gate leakage.

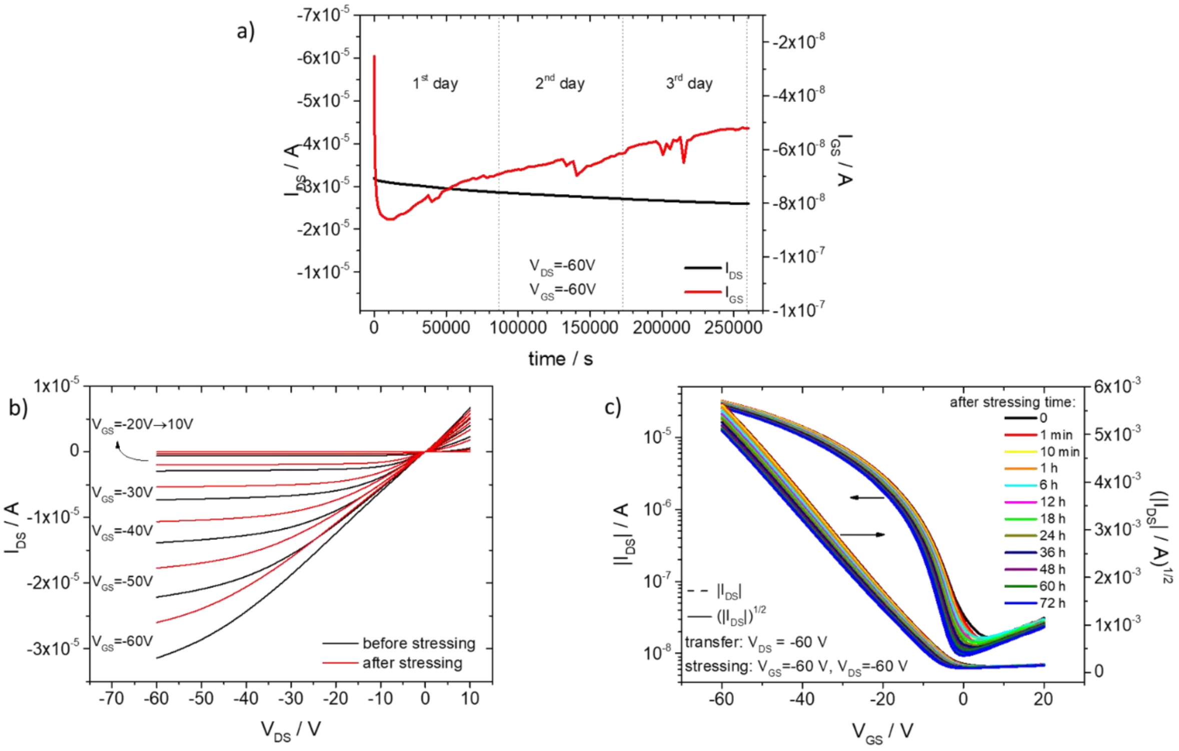

Figure S6. Operational stability tests of OFETs based on SVA TIPS-pentacene/366 kDa PS blend: a) I_{DS} and I_{GS} as a function of stressing time, b) output characteristics measured before and after 72hrs of stressing, c) transfer characteristics for different stressing times.

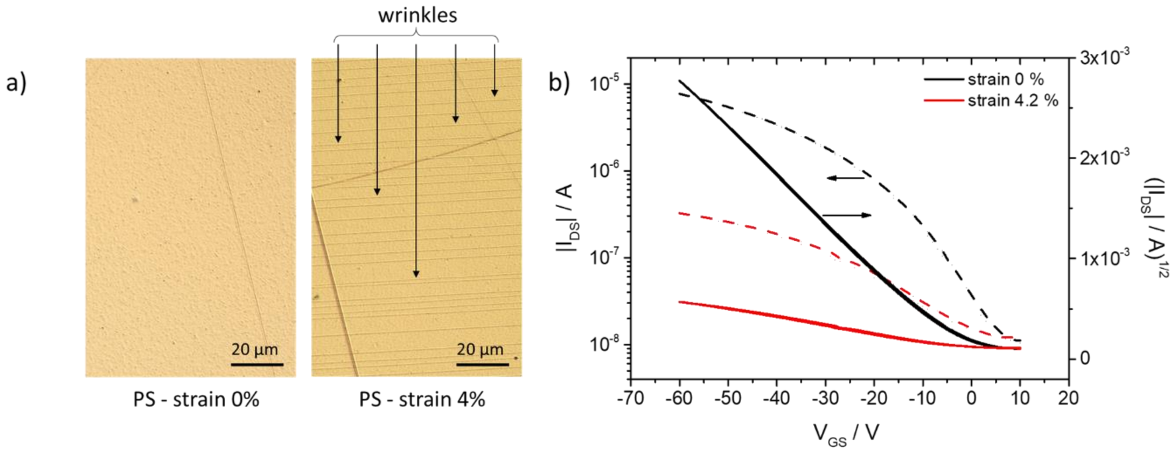

Figure S7. a) Microscope images of neat PS film spin-cast on a gold gate electrode before and after applying 4% strain, wrinkles lines are caused by elongation of the film beyond the yield stress of PS; b) transfer characteristics of an OFET based on SVA TIPS-pentacene/PS blend before and after applying 4.2% strain.

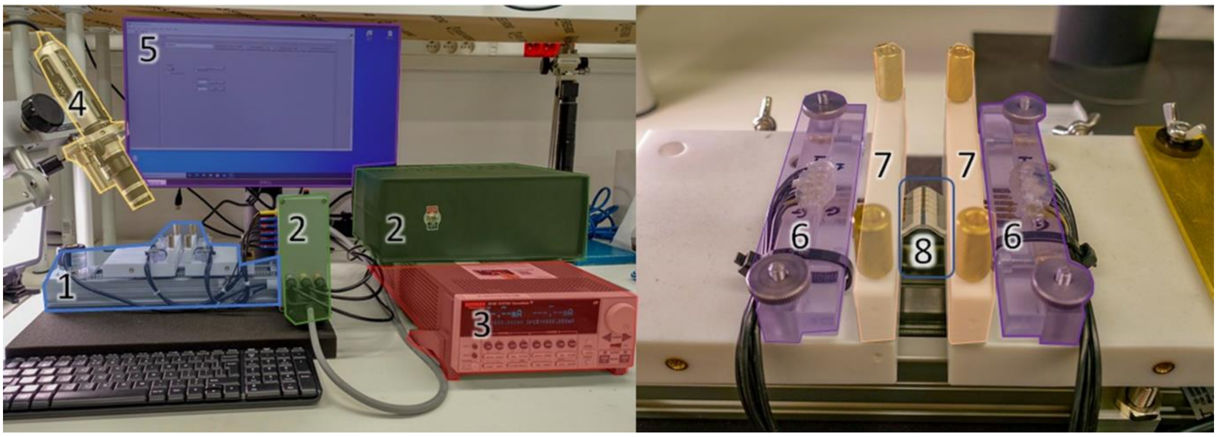

Figure S8. Bending measurement setup: complete setup with 1) bending device, 2) electronic control module, 3) Keithley 2634B source meter, 4) digital microscope, 5) control computer; a close-up image of the bending device: 6) contact electrode array, 7) securing clamps, 8) bent sample consisting of 5 OFET devices.

The transistor samples were prepared on Kapton® foil which improved the handling and increased the applied strain on the semiconducting layer. By choosing higher thicknesses of the support foil it was possible to apply higher amounts of strain to the sample. In this design both clamps (Figure S8) can be moved simultaneously what ensures that the bent sample remains in the same position and the forces applied to the devices are uniformly distributed. A stationary optical microscope was used to take precise photographs of the bent area. The images were then analyzed with calibrated software in order to determine precisely the bend radius. Following this approach, it is possible to precisely determine the strain at any position of the clamps for different foil materials and thicknesses. To ensure a low signal-to-noise ratio of the measurement several steps were taken to isolate each of the transistor signals and to

ensure stable contact of the electrodes. Each sample consisted of 5 OFETs in bottom gate, top contact configuration with channel length of 30 µm and width of 1 mm. Electrical measurements were conducted in open air and room temperature conditions. Transistor characterization was performed by using a Keithley 2634B source meter connected to the bending setup, under a drain bias of -60 V and gate biases varied from 10 V to -60 V.

Transistors were studied on the bending setup in Figure S8. The OFET devices were firstly measured at their flat position, bias stress measurement was performed and then after each decreasing bending radius a full measurement sweep was performed.

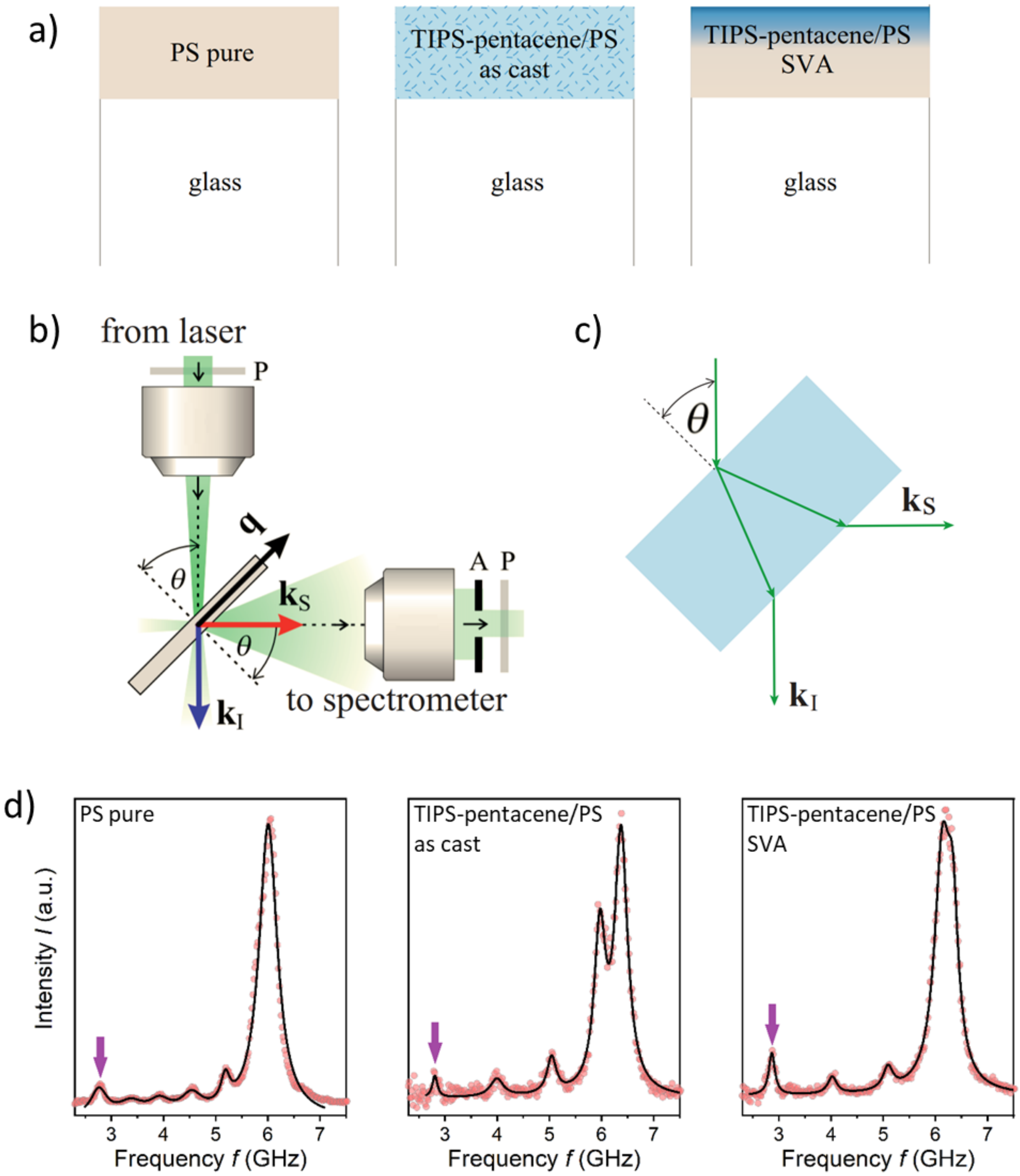

Figure S9. BLS – experimental, a) Schematic side view of samples: PS (left), TIPS-pentacene/PS blend (middle), phase-separated TIPS-pentacene/PS (right). b) Scheme of the laser beam passing through the sample, where symbols A and P denote aperture and polarizer, respectively. c) Scheme of the BLS scattering transmission geometry, where *ki* and *ks* represent the wave vectors of the incident and scattered light, respectively. 2ϑ is the scattering angle, d) BLS spectra (red points) of PS, amorphous TIPS-pentacene/PS, and phase-separated TIPS-pentacene/PS with M_w of 366 kDa. Data were analyzed with the Lorentz

function representation (black lines). Purple arrows indicate peaks with the lowest frequency f_R corresponding to the Rayleigh surface acoustic wave (RSW).

a)

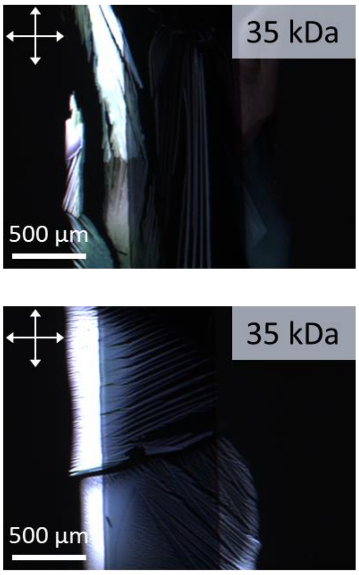

b)

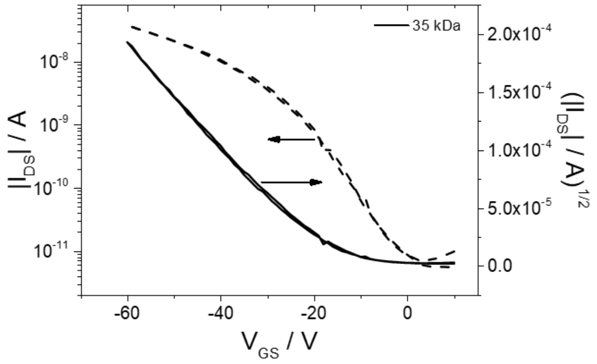

PS Mw	$V_{th\text{-}aver.}$ [V]	μ_{MAX} [$cm^2V^{-1}s^{-1}$]	$\mu_{average}$ [$cm^2V^{-1}s^{-1}$]
35 kDa	18	$3 \cdot 10^{-4}$	$7 \cdot 10^{-5}$

Figure S10. a) Polarized optical microscope images of SVA TIPS-pentacene/35 kDa PS blend film and b) transfer characteristics at V_{DS} = -60 V of OFET based on SVA TIPS-pentacene/35 kDa PS blend film and its operation parameters.

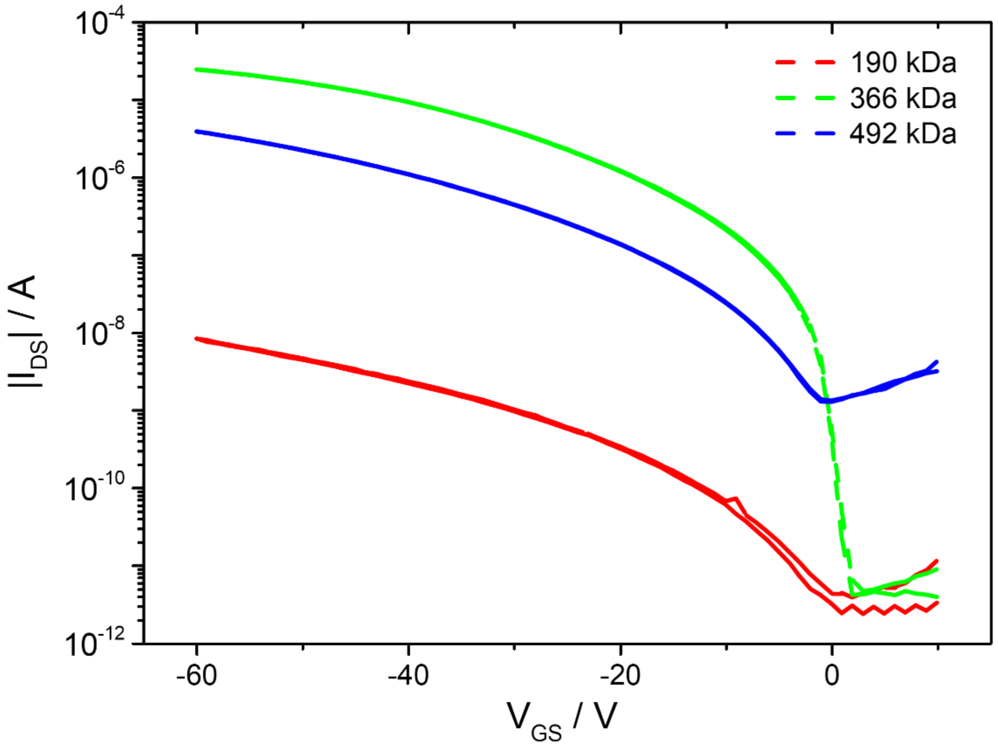

Figure S11. Transfer characteristics at V_{DS} = -60 V of OFET based on SVA TIPS-pentacene/ blend films with different M_w of PS blend film and its parameters with backsweep curves.

Table S1. Values of the frequency f_{R}, phase velocity v_{R}, transverse sound velocity v_{t}, longitudinal sound velocity v_{l} and Young's modulus E for three samples: PS, amorphous TIPS-pentacene/PS and TIPS-pentacene/PS phase-separated (366 kDa). The value of Young's modulus for Tips-pentacene before phase-separation was calculated from Wood's law.

	f_{R} [GHz]	v_{R} [ms^{-1}]	v_{t} [ms^{-1}]	v_{l} [ms^{-1}]	E [GPa]
PS	2.76	1037.31	1114.78	2166.75	3.44
TIPS-pentacene/PS amorphous	2.80	1054.85	1133.64	2203.40	3.58
TIPS-pentacene/PS phase-separated	2.86	1077.28	1157.74	2250.24	3.73

To determine the Young's modulus *E* of the films, we employed Brillouin light scattering (BLS) [S1] in the VV (vertical polarization of the incident and scattered light) transmission geometry configuration (Figure S9 b, c) [S2]. Measurements were performed at room temperature using six-pass tandem Fabry-Perot interferometer and 532 nm CW laser as the incident light source. The scheme of the laser beam passing through the sample and the scattering geometry are shown in Figure S9 b and c, respectively. The difference between scattered $\boldsymbol{k}_s$ and incident $\boldsymbol{k}_i$ light is defined as scattering wave vector:

$$\pm q = k_s - k_i \qquad (1)$$

and has a magnitude:

$$q = \frac{4\pi}{\lambda}\sin\theta \qquad (2)$$

in transmission geometry, where: λ – the wavelength of incident light in vacuum (CW laser with $\lambda = 532$ nm), 2θ – scattering angle. All measurements were conducted at fixed $q = 0.0167$ nm^{-1} ($2\theta = 90°$). The peak frequencies from the experimental spectra give access to the value of the longitudinal and transverse sound velocities. This allows to calculate values of the Young's modulus and, in combination with the finite element method (FEM), to estimate the sample thickness. Figure S9 a shows schematics of the samples: thin films of i) polystyrene (PS), ii) amorphous TIPS-pentacene/PS blend, and iii) phase-separated TIPS-pentacene/PS after SVA on a thick auxiliary glass substrate. Figure S9 d displays typical BLS spectra for thin films, with discrete acoustic modes [S3], for three samples: PS, amorphous TIPS-pentacene/PS, and phase-separated TIPS-pentacene/PS with M_w of 366 kDa. All spectra were obtained at the scattering angle $2\theta = 90°$ what corresponds to the acoustic wave number q=0.0167 nm^{-1}.

The frequency of the lowest peak f_{R} (purple arrows in Fig. 2) is assigned to the Rayleigh surface wave (RSW) [S4] propagating in the film. This assumption is justified by the wavelength of

RSW, which is over twice shorter than the thickness of the film. Phase velocity v_{R} for the f_{R} is calculated from the equation:

$$v_{\mathrm{R}} = \frac{2\,\pi\, f_{\mathrm{R}}}{q} \qquad (3)$$

RSW velocity v_{R} is always lower than transverse sound velocity v_{t} by a factor of ξ, $v_{\mathrm{R}} = \xi\; v_{\mathrm{t}}$, which is a characteristic value for a given medium described by the equation [S5]:

$$\xi^6 - 8\,\xi^4 + 8\,\xi^2 \left[3 - 2\left(\frac{v_{\mathrm{t}}}{v_{\mathrm{l}}}\right)^2\right] - 16\left[1 - \left(\frac{v_{\mathrm{t}}}{v_{\mathrm{l}}}\right)^2\right] = 0 \qquad (4)$$

The ratio of transverse and longitudinal sound velocity is given by:

$$v_{\mathrm{t}}/\,v_{\mathrm{l}} = [(1 - 2\,\nu)/2\,(1 - \nu)]^{1/2} \qquad (5)$$

where: ν – Poisson's ratio. Including $\nu = 0.32$, ξ equals 0.93. For isotropic media, v_{l} and v_{t} depend on the mass density ρ and two independent components of the elastic tensor C_{11} and C_{44}, which are described by the Lame parameters λ and μ. It is known that elastic constants are all related. Relation of Lame parameters with Young's modulus and Poisson's ratio are as follows:

$$v_{\mathrm{l}} = \sqrt{\frac{E\,(1-\nu)}{\rho\,(1+\nu)(1-2\,\nu)}},\; v_{\mathrm{t}} = \sqrt{\frac{E}{2\,\rho\,(1+\nu)}} \qquad (6)$$

where: ρ – mass density. $\rho_1 = 1.05$ g/cm^3, $\rho_2 = 1.104$ g/cm^3, $\rho_3 = 1.055$ g/cm^3 for PS, Tips-pentacene, blend of PS and Tips-pentacene were used, respectively. Table X shows the calculated values of sound velocities v_{l}, v_{t}, and Young's modulus E for PS, amorphous TIPS-pentacene/PS, and phase-separated TIPS-pentacene/PS (M_w of 366 kDa). The missing value of Young's modulus for the semiconductor was calculated based on Wood's law [S6] for the mixture:

$$E = \left(\frac{\beta}{E_1} + \frac{1-\beta}{E_2}\right)^{-1} \qquad (7)$$

where: $E = 3.57$ GPa – Young's modulus for the mixture of PS and Tips-pentacene, E_1 – Young's modulus for Tips-pentacene, $E_2 = 3.44$ GPa – Young's modulus for PS, $\beta = 0.07$ – volume fraction.

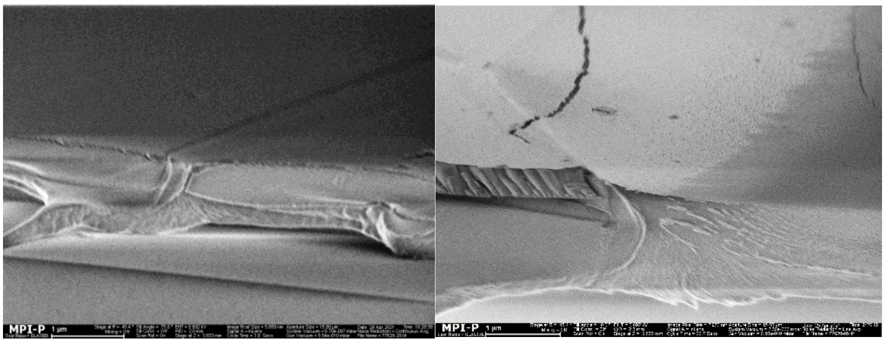

Figure S12. The cross-section SEM image of SVA TIPS-pentacene/366 kDa PS blend without graphic processing.

References

[S1] Brillouin, L. Diffusion de la lumi'ere et des rayons X par un corps transparent homogene *Ann. Phys.* **1922**, *9* (17), pp. 88–122. DOI: 10.1051/anphys/192209170088

[S2] Speziale, S.; Marquardt, H.; Duffy, T. Brillouin Scattering and its Application in Geosciences *Rev. Mineral. Geochem.* **2014**, *78* (1), 543-603. DOI: 10.2138/rmg.2014.78.14

[S3] Carlotti, G. Elastic Characterization of Transparent and Opaque Films, Multilayers and Acoustic Resonators by Surface Brillouin Scattering: A Review *Appl. Sci.* **2018**, *8*, 124. DOI: 10.3390/app8010124

[S4] Lord Rayleigh, D.C.L. F.R.S. On Waves Propagated along the Plane Surface of an Elastic Solid *Proc. London Math.* **1885**, *17*, 4-11. DOI: 10.1112/plms/s1-17.1.4

[S5] Landau, L.D.; Lifshitz, E. M. Theory of Elasticity *Course of Theoretical Physics* **1984**, *7*, 3rd edition, Institute of Physical Problems, USSR Academy of Sciences. ISBN: 9780750626330

[S6] Reig, D.S.; Hummel, P.; Wang, Z.; Rosenfeldt, S.; Graczykowski, B.; Retsch, M.; Fytas, G. Supporting information of Well-defined metal-polymer nanocomposites: The interplay of structure, thermoplasmonics, and elastic mechanical properties *Phys. Rev. Materials,* **2018**, *2*, 123605. DOI: 10.1103/physrevmaterials.2.123605
